# A Novel Image Analysis Approach Reveals a Role for Complement Receptors 1 and 2 in Follicular Dendritic Cell Organization in Germinal Centers

**DOI:** 10.3389/fimmu.2021.655753

**Published:** 2021-04-12

**Authors:** Jessica C. Anania, Annika Westin, Jeremy Adler, Birgitta Heyman

**Affiliations:** ^1^ Department of Medical Biochemistry and Microbiology, Uppsala University, Uppsala, Sweden; ^2^ Department of Immunology, Genetics and Pathology, Facilities, BioVis, Uppsala University, Uppsala, Sweden

**Keywords:** follicular dendritic cell (FDC), germinal center (GC), complement receptor, Fc receptor, immune complex (IC), ImageJ macro, IgM receptor

## Abstract

Follicular dendritic cells (FDCs) are rare and enigmatic cells that mainly reside in germinal centers (GCs). They are capable of capturing immune complexes, *via* their Fc (FcRs) and complement receptors (CRs) and storing them for long periods in non-degradative vesicles. Presentation of ICs on FDCs to B cells is believed to drive affinity maturation. CR1 and CR2 are expressed on B cells and FDCs. Cr2 knock out (KO) mice, lacking both receptors, have impaired antibody and GC responses. Utilizing a novel ImageJ macro to analyze confocal fluorescence microscopy images of spleen sections, we here investigate how FDCs in wild type (WT) and Cr2 KO mice behave during the first two weeks after immunization with sheep red blood cells (SRBC). Mice were immunized with SRBC i.v. and spleen and serum samples harvested at various time points. As expected, antibody and GC responses in Cr2 KO mice were impaired in comparison to WT mice. Fewer FDCs were identified in Cr2 KO mice, and these exhibited differential localization and organization in comparison to WT mice. WT FDCs were primarily located within GCs at the light zone/dark zone border. FDCs from WT but not Cr2 KO mice were actively dispersed in GCs, i.e. tended to move away from each other, presumably to increase their surface area for B cell interaction. FDCs from Cr2 KO mice were more often found on follicles outside of the GCs and those within the GCs were closer to the periphery in comparison to WT FDCs. Expression of CR1 and CR2, FcγRIIB, and FcµR increased in FDCs from WT mice during the course of immunization. The results suggest that decreased ability to capture ICs by FDCs lacking CR1 and CR2 may not be the only explanation for the impaired GC and antibody responses in Cr2 KO mice. Poor FDC organization in GCs and failure to increase receptor expression after immunization may further contribute to the inefficient immune responses observed.

## Introduction

Follicular dendritic cells (FDCs) constitute a highly specialized cell population primarily residing within germinal centers (GCs), both in secondary lymphoid organs and so-called tertiary lymphoid organs, which form in inflamed tissues (“ectopic GCs”) (1–3). Unlike other immune cells, FDCs are not derived from hematopoietic stem cells in the bone marrow but from ubiquitous mural perivascular precursors (4). In spite of their name, they are not related to conventional dendritic cells, do not express MHC-II, and consequently do not present peptides to T cells. However, FDCs in the GC light zone (LZ) are capable of capturing antibody and complement immune complexes (ICs) by their wide variety of Fc (FcR) and complement (CR) receptors, including FcγRIIB (CD32B), FcϵRII (CD23), Fcα/μR (CD351), CR1(CD35) and CR2 (CD21) (5–12). Display of antigens on the surface of FDCs is believed to serve as the first selection step for GC B cells having undergone somatic hypermutation (3, 7, 13). Recent studies have shown that FDCs internalize ICs through endocytosis into non-degradative vesicles, which are periodically recycled to the surface to allow interaction with GC B cells (12). This can take place for long periods, even years (14). Thereby FDCs can act as “antigen libraries” and play an important role in antibody affinity maturation and the formation of immune memory (1). FDCs can be divided into two subsets, LZ and dark zone (DZ) FDCs (3, 9). The fore mentioned LZ FDCs express FcRs and CRs and can be characterized by high expression of CXCL13. DZ FDCs express CXCL12 and low or no FcRs and CRs, and were shown to be necessary for GC CXCL12 cytokine gradients in the DZ (9). Complement receptors 1 and 2 (CR1/2) were expressed by both FDC subsets although expression was higher in LZ FDCs.

In the absence of complement components C1, C2, C3, or C4, as well as CR1/2, antibody responses are strongly impaired [reviewed in (15, 16)]. The molecular mechanism behind this is not entirely clear. Most likely, ligation of CR1/2 is of central importance since lack of C1, C2, and C4 leads to poor generation of the C3 split products iC3b, C3dg, and C3b which are the ligands for these receptors. In mice, CR1 and CR2 are splice forms of the same gene, *Cr2* (17), and most studies of the influence on immune responses have been performed in Cr2 KO mice which lack both receptors. Murine CR1/2 are expressed on B cells and FDCs (18), and therefore a role for either or both cell types may be envisaged to explain the importance of complement in the generation of antibody responses. FDC involvement could be caused by the capture and presentation to B cells of ICs. B cell involvement could be explained by enhanced B-cell signaling, resulting from co-crosslinking of the CR2/CD19/CD81 coreceptor complex and BCR (19, 20). Alternatively, B cell involvement may be explained by enhanced transport of ICs from the marginal zone (MZ) to B cell follicles by MZ B cells. These cells express high levels of CR1/2 and shuttle between the MZ and the follicle, delivering antigen to FDCs (21–23). In addition to an impaired antibody response, CR1/2-deficiency has been shown to result in a marked decrease in size and/or number of GCs (24–27). The relative role of CR1/2 expression by FDCs versus B cells for normal antibody responses has been analyzed in bone marrow chimeric mice. In many reports, expression of the receptors on FDCs was shown to play an important role (25, 26, 28–30) although parallel expression on B cells was sometimes required for full restoration of the antibody response (25, 28). In a few studies, a major role for CR1/2 on B-cells was reported (24, 27, 31). These discrepancies are most likely explained by the use of different antigens and experimental systems.

We were interested in determining how normal splenic FDCs, which have been far less studied than lymph node FDCs, responded to antigen during the first two weeks after an immunization and whether expression of CR1/2 impact FDC behavior. SRBC was chosen as antigen because it can be administered i.v. without adjuvants and still induce a strong CR1/2-dependent antibody and splenic GC response. Using a novel ImageJ macro, confocal microscopy images of spleen sections were analyzed mathematically and the organization of FDCs within GCs in WT and Cr2 KO mice, as well as expression of various complement and FcRs, were determined.

## Methods

### Animals

Wild type (WT) BALB/c mice were from Bommice, Ry, Denmark. Cr2 knock out (Cr2 KO) mice, a gift from Hector Molina (32), were backcrossed to BALB/c for 10 generations. Mice within each experiment were matched for age (6-10 weeks) and sex. Animals were bred and maintained in the animal facilities at the National Veterinary Institute (Uppsala, Sweden).

### Immunization and Blood Sampling

Sheep red blood cells (SRBC) were obtained from Håtunalab AB (Håtunaholm, Sweden) and were stored in sterile Alsever’s solution at 4°C. SRBC was prepared at 5 × 10^8^/ml in PBS and 100 µl i.v. immunizations were done in one of the lateral tail veins. To analyze antibody and GC responses, serum samples and spleens were collected from unimmunized mice (day 0) and mice immunized 1, 3, 6, or 14 days before. Mice were bled into individual tubes and the blood allowed to clot at 4°C overnight. Sera were then removed, spun at 13,000 rpm, cell-free fractions collected and frozen at −20°C until use in ELISA.

### Antibodies

Goat anti-mouse IgM alkaline phosphatase (AP) (Jackson ImmunoResearch Laboratories,115-055-020) and sheep anti-mouse IgG AP (Jackson ImmunoResearch Laboratories, 515-055-071) were used for anti-SRBC ELISA.

The following panel of antibodies were used for microscopy experiments. Biotinylated anti-mfge8 (FDC-M1, in house) prepared using EZ-Link Sulfo-NHS-LC-Biotinylation Kit (ThermoFisher Scientific, 21435), BV421 streptavidin (BD Biosciences, 563259), unconjugated rat anti-mouse CD35 (CR1) (clone 8C12, in house), eFluor 660 mouse anti-rat IgG2a (clone r2a-21B2) (ThermoFisher Scientific, 25-0232-82), phycoerythrin (PE) rat anti-mouse CD169 (seglec-1/MOMA) (clone 3D6.112) (BioLegend, 142404), FITC anti-mouse CD21/35 (CR1/2) (clone 8D9) (ThermoFisher Scientific, 11-0211-82), FITC rat anti-mouse CD16/32 (FcγRIII/FcγRIIB) (clone 93) (BioLegend, 101305), Alexa Fluor 488 mouse anti-human FcµR (MM3) (R&D Systems, FAB9494G-100UG), allophycocyanin (APC) rat anti-human/mouse CD45R (B220) (clone RA3-6B2) (BD, 553092), Pacific Blue rat anti-mouse CD45R (B220) (clone RA3-6B2) (BD, 558108), eFluor 660 anti-mouse/rat Ki67 (clone SolA15) (Invitrogen, 50-5698-82), PE rat anti-mouse T and B cell activation antigen (GL7) (BD, 561530), lectin peanut agglutinin (PNA) from *Arachis hypogaea* (peanut) Alexa Fluor 488 Conjugate (ThermoFisher Scientific, L21409).

The following antibodies were used for flow cytometry experiments. Biotinylated anti-FDC-M1 and hamster anti-mouse ICAM-1 (CD54) (clone 3E2B) (ThermoFisher Scientific, Invitrogen, MA5405) were used as primary antibodies. Secondary flow cytometry antibodies were APC Cyanine7 streptavidin (BD Biosciences, 554063), Alexa Fluor 545 goat anti-hamster IgG (H+L) (ThermoFisher Scientific, Invitrogen, A21111), BV480 rat anti-mouse CD45 (BD, 566095), and Alexa Fluor 594 mouse anti-human FcµR (clone 992338) (R&D Systems, FAB9494T-100UG).

### Anti-SRBC ELISA

The ELISA used to detect IgG and IgM anti-SRBC has been described previously (33), the only difference being that we here analyzed IgG responses with anti-IgG antisera (and not anti-IgG-allotype antisera). Briefly, ELISA plates were coated with SRBC and incubated with appropriately diluted sera from immunized mice. After washing, Abs recognizing mouse IgM or IgG, conjugated to alkaline phosphatase were added (see antibody section for details). Absorbance at 405 nm was measured after incubation for 60 minutes.

### Confocal Microscopy

Spleens were harvested and fixed in 4% paraformaldehyde (PFA) (Electron Microscopy Sciences, 15710) for 30 minutes and subsequently mounted in optimal cutting temperature (OCT) compound (VWR BHD chemical, 361603E), snap frozen using liquid nitrogen, and stored at -80°C. Samples were then sectioned 10 µm thick using a Thermo Scientific CryoStar Nx70 Cryostat (Thermo Scientific) onto Superfrost Plus microscope slides (ThermoScientific, J1800AMNZ). The samples were stored at -80°C until fluorescence microscopy staining. Briefly, the spleen sections were rehydrated in phosphate-buffered saline (PBS) and blocked in 2% bovine serum albumin (BSA) (Sigma, A3912-50G) for 30 minutes at room temperature prior to staining. Antibodies were diluted in 2% BSA-PBS and incubated for 1 hour at room temperature. Samples were washed twice for 5 minutes in PBS between each staining step. Post staining slides were washed and mounted with Fluoromount G (ThermoFisher Scientific, 00-4958-02).

**Figure 1:**
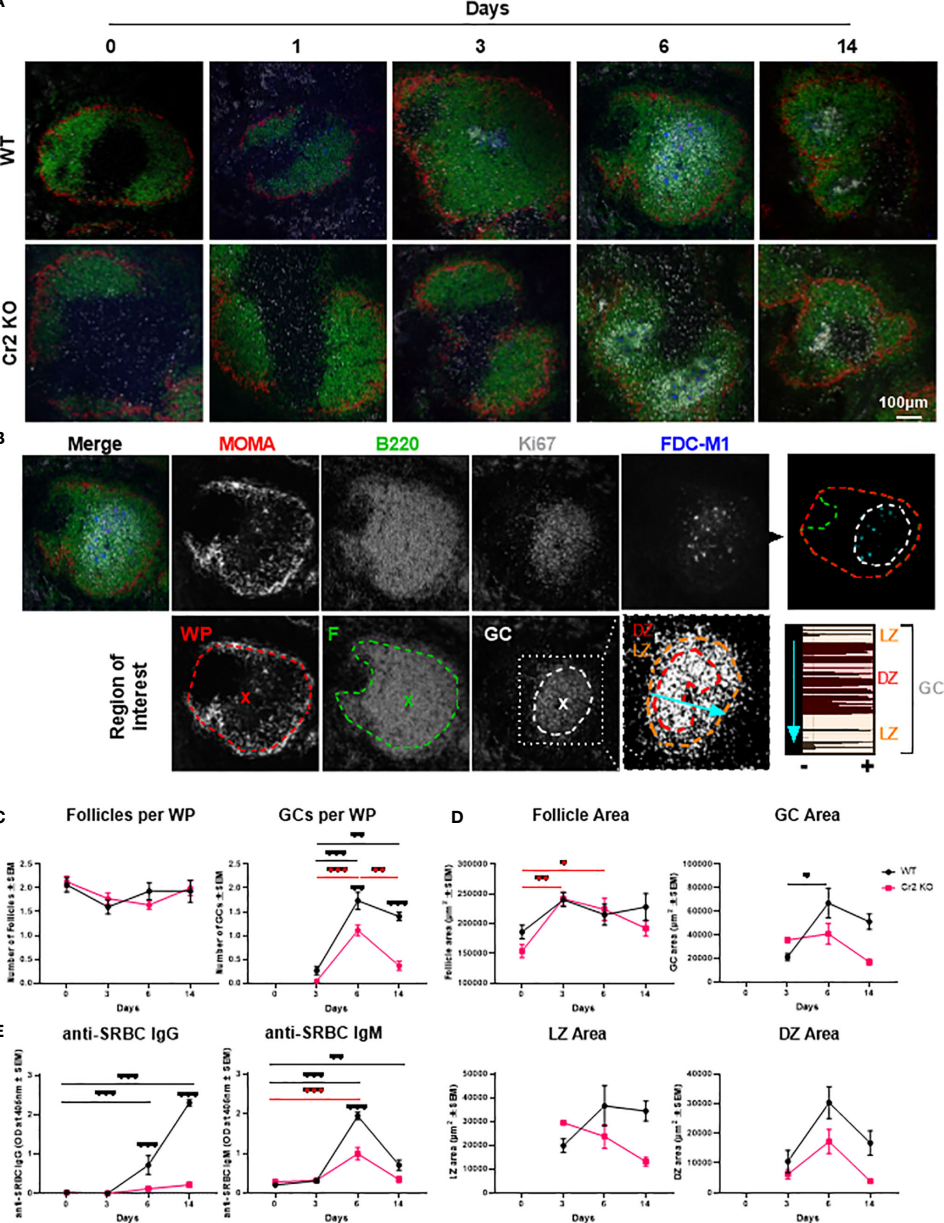
Cr2 KO mice have impaired GC and antibody responses. WT or Cr2 KO mouse spleens and sera were harvested from unimmunized mice (day 0) and from mice immunized with 5 x 10^7^ SRBC 3, 6, or 14 days before and sera and sections prepared as described in *Materials and Methods*. **(A)** Representative images of spleen WP regions surrounded with MZ regions (MOMA, red), B cell follicles (B220, green), GCs (Ki67, grey) and FDCs (FDC-M1, blue). **(B)** Example of day 6 WT image, illustrating regions of interest (ROIs) defined as per example image with each WP ROI defined as the area surrounded by MOMA (red), follicle (F) ROI as B220+ (green) and GC ROI as the Ki67+ (grey) areas, with the center indicated by “x”. GC ROIs were further divided into LZ and DZ based on the density of Ki67 within GC ROIs as highlighted by LineScan of line illustrated by light blue arrow. **(C)** The average number (± SEM) of B cell follicles and GC regions per white pulp were quantified for WT (black) and Cr2 KO (red) mice. **(D)** The area of follicle, GC, LZ and DZ ROIs (µm^2^ ± SEM). **(E)** IgM and IgG anti-SRBC responses in Cr2 KO mice. Sera were analyzed for IgM or IgG anti- SRBC *via* ELISA (optical density (O.D.) 405 nm ± SEM). Statistical differences between the groups were determined by two-way ANOVA. Statistical differences between WT and KO mice at each time point is shown above the upper curve and statistical differences between naive and immunized respective mice over the 14 day time course are indicated above horizontal lines (WT= black; KO= red). ***p values < 0.001, ** < 0.01, and * < 0.05. The data shown are 6-9 mice per group pooled from three independent experiments.

For confocal studies, two non-consecutive sections from each mouse spleen were stained and 5 white pulp (WP) regions/mouse analyzed. To prevent bias, WP regions were selected based on MZ morphology (i.e. MOMA staining) only. All other channels remained blinded post-acquisition setup, preventing biased selection of e.g. follicles with or without GCs. The data are based on 30-45 images, from 6-9 mice in each group. To quantify follicles, GCs, and FDCs, and to investigate FDC localization and organization (**Figures 1** **–** **4**), sections were stained with MOMA-PE, FDC-M1-biotin + Streptavidin-BV421, Ki67-e660, and B220-A488. To investigate FDC receptor expression (**Figures 5**, **6**), sections were stained with MOMA-PE, rat anti-mouse-CR1 + anti-rat IgG e660, and FDC-M1-biotin + Streptavidin-BV421 to identify FDCs in WP regions of interest (ROIs). Subsequently, staining for receptors was added to the FDC staining panel described above: CR1/2-FITC, FcγRIIB-FITC, or FcµR-FITC. In FDC receptor expression staining protocols, slides were blocked with 20% horse serum (Sigma, H1270-100ML) after staining with rat anti-mouse-CR1 + anti-rat IgG e660, i.e. before continuing with staining for FDCs and receptors.

**Figure 2:**
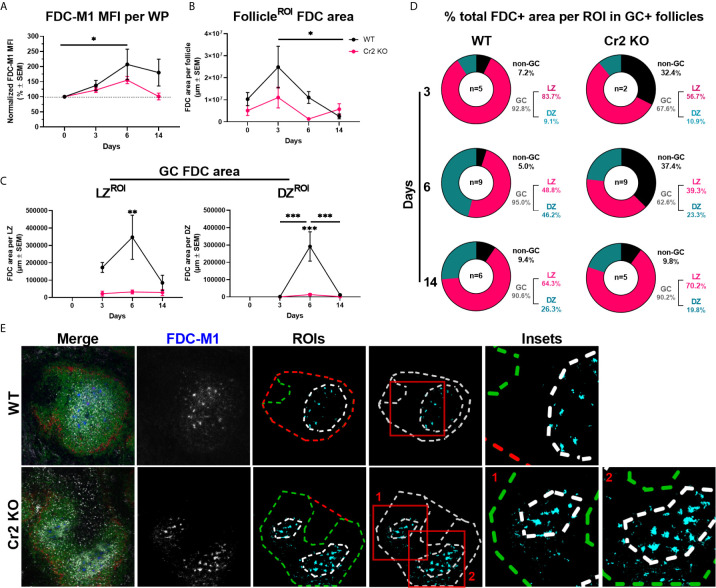
Cr2 KO mice have fewer FDCs in GCs than WT mice. WT or Cr2 KO mouse spleens were harvested from unimmunized mice (day 0) and from mice immunized with 5 × 10^7^ SRBC 3, 6, or 14 days before and prepared for confocal microscopy. **(A)** FDCs in the WP ROIs of WT (black) and KO (red) mice were quantified by FDC-M1 mean fluorescence intensity (MFI ± SEM). **(B)** Area of all FDCs present in each B cell follicle (µm^2^ ± SEM). **(C)** Area of FDCs within GC LZ (left) and DZ (right) regions (µm ± SEM). **(D)** Percentage of total GC+ follicle FDCs in each ROI 3-, 6- and 14-days post immunization. **(E)** Representative images of day 6 post immunization WT and Cr2 KO spleen WP regions surrounded with MZ regions (MOMA, red), B cell follicles (B220, green), GCs (Ki67, grey) and FDCs (FDC-M1, blue). ROIs of example images are outlined in middle panels to highlight distribution of GC and non-GC FDCs. Statistical differences between the groups in **(A–C)** were determined by two-way ANOVA. Statistical differences between WT and Cr2 KO mice at each time point is shown above the upper curve and statistical differences between naive and immunized respective mice over the 14 day time course are indicated above horizontal lines (WT= black; KO= red). ***p values < 0.001, ** < 0.01, and * < 0.05. The data shown are 6-9 mice per group pooled from three independent experiments, unless indicated (as in **D**).

**Figure 3:**
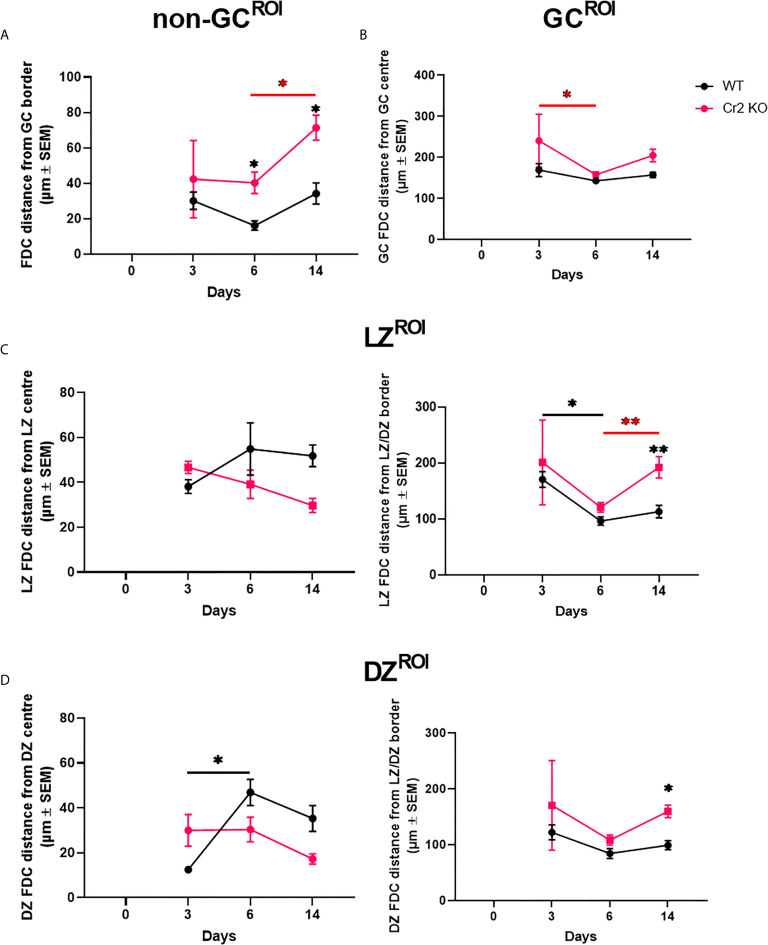
Expression of CR1/2 is needed for directing FDCs to GCs. WT or Cr2 KO mouse spleens were harvested from unimmunized mice (day 0) and from mice immunized with 5 x 10^7^ SRBC 3, 6, or 14 days before and sera and sections prepared as described in *Materials and Methods*. FDC localization within different WP regions was measured from the center or edge of each region. **(A)** Mean distance of follicle non-GC FDCs measured from the GC border (µm ± SEM). **(B)** Mean distance of all GC FDCs measured from the GC center (µm ± SEM). **(C)** Mean distance of LZ FDCs measured from the LZ center (left) and LZ/DZ border (right) (µm ± SEM). **(D)** Mean distance of DZ FDCs measured from the DZ center (left) and LZ/DZ border (right) (µm ± SEM). Statistical differences between the groups were determined by two-way ANOVA. Statistical differences between WT and KO mice at each time point is shown above the upper curve and statistical differences between naive and immunized respective mice over the 14 day time course are indicated above horizontal lines (WT= black; KO= red). p values ** < 0.01, and * < 0.05. The data shown are 6-9 mice per group pooled from three independent experiments.

**Figure 4:**
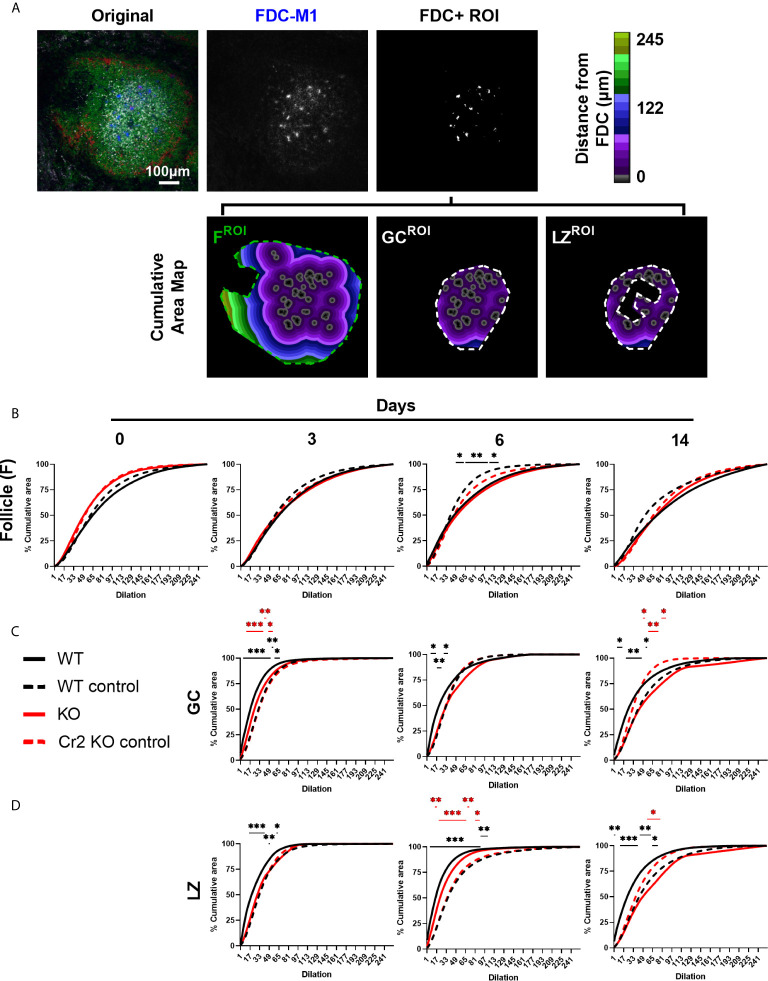
Cr2 KO FDCs show different organization to WT FDCs in follicles, GCs, and LZ. WT or Cr2 KO mouse spleens were harvested from unimmunized mice (day 0) and from mice immunized with 5 x 10^7^ SRBC 3, 6, or 14 days before and sera and sections prepared as described in *Materials and Methods*. **(A)** Representative image of WT mice day 6 with follicle (F), GC and LZ ROIs shown. Organization of FDCs (FDC-M1, blue) positive thresholded areas within **(B)** B cell follicle (B220, green), **(C)** GC (Ki67, all grey) and **(D)** LZ (Ki67, less dense grey) ROIs were evaluated by plotting mean percentage cumulative area within respective areas (as illustrated in **Supplementary Figure 1**). FDC+ regions for each image, randomly distributed within each ROI, served as controls (n=8 per sample image, broken line). Statistical differences between the groups were determined by two-way ANOVA. Statistical differences between experimental sample and randomized controls for respective strains at each time point are shown above the upper curve and statistical differences between naive and immunized respective mice over the 14 day time course are indicated above horizontal lines (WT = black; KO = red). ***p values< 0.001, ** < 0.01, and * < 0.05. The data shown are 6-9 mice per group pooled from three independent experiments.

**Figure 5:**
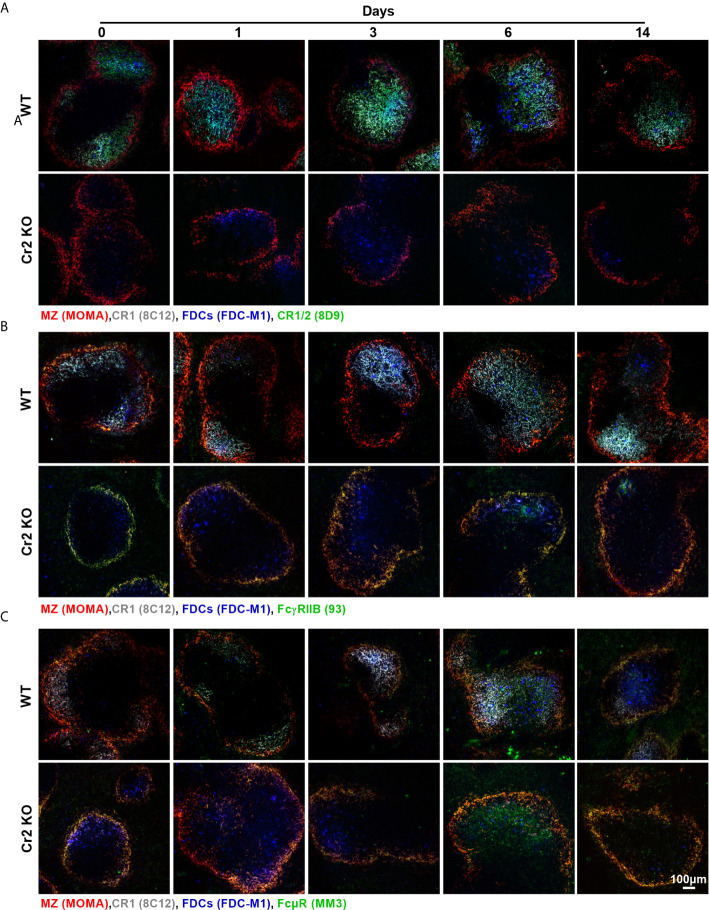
Representative images of CR1/2, FcγRIIB, and FcµR expression in WP of WT and Cr2 KO mice. Representative images of spleens from unimmunized mice (d 0) or from mice immunized 1-14 days before. WP regions, surrounded by metallophilic macrophages (MOMA, red) indicating MZ regions, FDCs (FDC-M1, blue), CR1 (8C12, grey) and immunoreceptors, **(A)** CR1/2 (8D9, green), **(B)** FcγRIIB (clone 93, green) and **(C)** FcµR (MM3, green) are shown.

**Figure 6:**
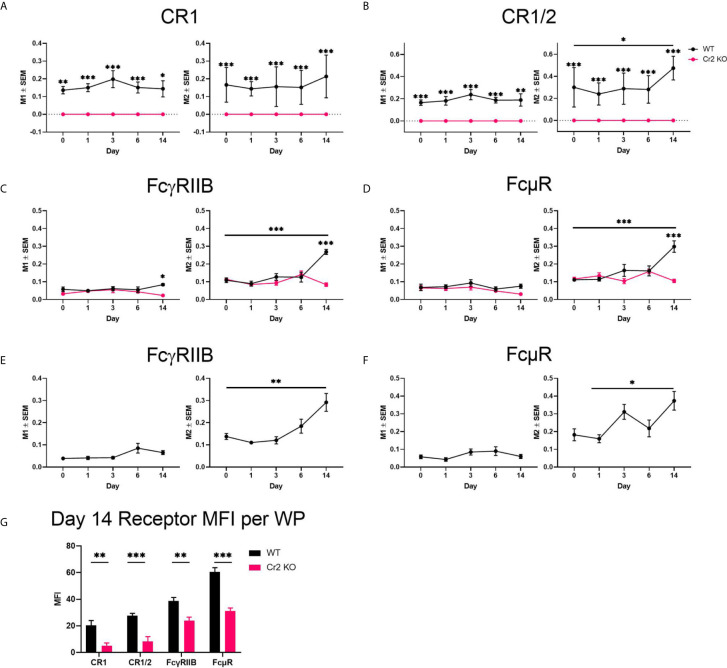
Expression of CR1/2, FcγRIIB, and FcμR increases in response to SRBC in WT mice. WT or Cr2 KO spleens were harvested from unimmunized mice (day 0) and from mice immunized with 5 × 10^7^ SRBC 1, 3, 6, or 14 days before and prepared for confocal microscopy Mander’s colocalization co-efficient of total whole image FDCs (FDC-M1) with **(A)** CR1 (8C12), **(B)** CR1/2 (8D9), **(C)** FcγRIIB (clone 93) and **(D)** FcµR (MM3). CR1 was also used as a comparative FDC marker for **(E)** FcγRIIB and **(F)** FcµR Mander’s colocalization. M1 is the frequency of FDCs where immunoreceptors are expressed. M2 is the frequency of respective immunoreceptor expression on FDCs. **(G)** CR1 (8C12), CR1/2 (8D9), FcγRIIB (clone 93) and FcµR (MM3) mean fluorescence intensity (MFI ± SEM) in WP ROIs of WT (black) and KO (red) mice 14 days post SRBC immunization. Statistical differences between the groups were determined by two-way ANOVA. Statistical differences between WT and KO mice at each time point is shown above the upper curve and statistical differences between naive and immunized respective mice over the 14 day time course are indicated above horizontal lines (WT = black; KO = red). *** p values< 0.001, ** < 0.01, and * < 0.05. The data shown are six to nine mice per group pooled from three independent experiments.

Spleen sections were imaged at room temperature using a Zeiss LSM 710 Elyra S.1, AxioObserver confocal microscope equipped with 405, 488, 561, and 633 nm lasers, and Plan-Apochromat 20 ×/0.8 M27. Single plane images were acquired using Zen (Black edition) software.

### Image Analysis

All images were analyzed using the open-source Java application ImageJ (https://imagej.nih.gov/ij/). Mander’s colocalization coefficients (M1 and M2) (34) were calculated using ImageJ coloc2 plugin, part of the analysis options of the expanded ImageJ version Fiji. M1 and M2 measure the fraction of the total fluorescence found in the presence of a second fluorophore within a ROI.

Region areas, radii, mean fluorescence intensity (MFI) and distance maps (**Figures 1**–**4**) were calculated using a custom macro outlined in **Supplementary Figure 1**. This ImageJ macro code is available in supporting data (https://github.com/J-C-Anania/Mapping-FDCs-in-GCs). Spleen sections of WT and Cr2 KO naive mice (day 0) and immunized mice at day 3, 6 and 14 were analyzed. Splenic structures were defined as regions of interest (ROIs) based on the following stains; WP (surrounded by MOMA+ metallophilic macrophages), B cell follicle (B220+ area), and GC (condensed Ki67+ area). This was achieved by thresholding the respective stains and selecting peripheral positive areas to be used to set the ROI perimeter. In other words, the macro defines condensed Ki67+ regions (via thresholding), creating a binary image of Ki67+ regions. The Ki67+ regions in the binary image are each assigned a different identification number (intensity value) by the macro allowing the user to select individual Ki67+ regions at the periphery of GC structures. The macro subsequently joins each of these selections, *via* convex hull process, to surround the selected region creating the GC ROI. Therefore, the macro relies on both automated computational processing and user input to select GC ROI.

GC ROIs were further divided into LZ and DZ regions based on Ki67 density. This was achieved by using erode functions that remove small areas while retaining larger ones, i.e. removing certain number of pixels from the edge of positive regions, to differentiate lower density positive regions such as the moderate density Ki67+ LZ from higher density Ki67+ positive DZ regions (Computational processing panels, **Supplementary Figure 1B**).

ROI area and radii (maximum radius from ROI center) were then calculated. FDC (FDC-M1+) areas and FDC-M1 MFI within WP ROIs were utilized to quantitate FDCs.

Distribution of FDC areas were subsequently mapped in each of these respective ROIs. ROI center or border coordinates were utilized to generate distance maps using Euclidean Distance Maps (EDMs) (35). EDMs increase in intensity as with each pixel from the starting point, which provides a measure of distance from the selected starting point. Herein, the Euclidean distance provides the straight-line distance between the ROI center or border to each FDC area,i.e. FDCs closer to the ROI center or border parameter selected will have a lower intensity than those further away. The mean EDM FDC distance was calculated for each image, and subsequently averaged per mouse. FDCs outside a selected ROI were assigned null values and values <1 excluded from analysis.

In **Figure 4**, the organization of FDC areas within these same ROIs (follicle, GC and LZ) was compared to random distribution controls by sequentially dilating (swelling) FDC areas and plotting the percentage of the ROI area filled after each dilation. Using the RandomJ ImageJ plugin, FDC areas were randomly distributed within the selected ROI eight times and averaged. These values were then compared to the biological (i.e. experimental) samples. If FDCs are clustered, the dilations from nearby cells quickly overlap and therefore the rate at which the image is filled is slow compared to the randomized control. If FDCs are actively dispersed, i.e. spread far apart, their separation delays the overlap and hence fills the ROI rapidly in comparison to the randomized control. All comparisons between randomized controls and biological samples are made within the same follicle, GC, or LZ ROI.

FcR and CR expression on FDCs were evaluated using Mander’s colocalization coefficients as detailed above (**Figures 6A–F**). Additionally, the overall receptor expression was quantified by measuring the MFI within WP ROIs (**Figure 6G**).

### Flow Cytometric Analysis of FDCs

FDCs were isolated from murine spleen samples using a spleen dissociation kit (MiltenyiBiotech, 130-095-926) on a gentileMACS dissociator (MiltenyiBiotec, 130-093-235). Samples were subsequently filtered (70 µm), RBCs lysed using ammonium-chloride-potassium (ACK) buffer and samples depleted for CD45 positive cells, using mouse CD45 MicroBeads (MiltenyiBiotec, 130-052-301) over MACS LS separation columns (MiltenyiBiotec, 130-042-401). Enriched CD45 negative splenocytes were subsequently stained for FDCs using biotinylated FDC-M1 and anti-ICAM-1, then washed and stained with secondary antibodies streptavidin-APC Cy7 and anti-hamster-Alexa561 in conjunction with CD45-BV480, and FcµR-Alexa591 in Brilliant Stain Buffer BD (Biosciences, 563794) (see antibody section for details). CD45 negative, FDC-M1+, ICAM-1+ FDCs were analyzed using BD FACSariaIII (Becton, Dickinson and Company).

### Statistical Analysis

The data in **Figures 1**–**6** were obtained from the same three independent experiments and pooled from 6-9 mice per group. For confocal studies, 5 WP regions from the two non-consecutive sections taken from each mouse, were analyzed and averaged per mouse prior to conducting statistical analysis. P values <0.05 were considered statistically significant. Statistical differences between the groups were determined by two-way ANOVA. p<0.001 is symbolized by ***; p<0.01 by **; and p<0.05 by *.

### Ethics Approval

This study was carried out in accordance with the recommendations of the Uppsala Animal Research Ethics Committee (permit numbers C25/15 and Dnr 5.8.18-02583/2018). The protocols were approved by the Uppsala Animal Research Ethics Committee.

## Results

### Cr2 KO Mice Have Impaired GC and Antibody Responses

In order to characterize ROIs within splenic WP, two non-consecutive spleen sections from each WT and Cr2 KO mice, immunized with 5 × 10^7^ SRBC i.v., were imaged by confocal microscopy (**Figure 1A**; single channels shown in **Supplementary Figure 2**). The different ROIs (WP, follicles, GC, DZ, and LZ) were defined as illustrated in **Figure 1B**, by thresholding images and selecting peripheral positive regions allowing the macro to surround the ROI.

The GC marker Ki67 was selected due to its ability to differentiate between the LZ and DZ (9, 36). LZ and DZ ROIs exhibit moderate and high density, respectively, as highlighted by the frequency of peaks in linescan analysis of the GC ROI (**Figure 1B**). Importantly, Ki67 localization was consistent with traditional GC markers such as GL7 and PNA (**Supplementary Figure 3**). No significant difference in GC area was observed between these GC markers, supporting selection of Ki67 as a reliable GC marker.

WP regions, per definition surrounded by metallophilic macrophages stained with MOMA, contained B cell follicles at all timepoints (**Figures 1A–C** **)**. Quantification of B cell follicles per WP showed no significant difference in numbers neither between the two strains nor over time (**Figure 1C**, left). Follicles did increase slightly in size over the 14 day time course, as indicated by an increase follicle area (**Figure 1D**, top left). However, no significant differences were observed between WT and Cr2 KO mice.

GCs were detectable from 3 days post SRBC immunization (**Figures 1A, C** **)**. WT mice had significantly more GCs than Cr2 KO mice (**Figure 1C**, right). While Cr2 KO mice were capable of forming GCs, they were much shorter lived, returning to approximately baseline numbers at day 14 (**Figure 1C**, right). The GC area was lower in Cr2 KO than in WT mice, 6 and 14 days post immunization, but the difference did not reach statistical significance (**Figure 1D**, top right). GC area was significantly increased at 6 days post immunization in comparison to day 3, before decreasing in area at day 14. LZ area measurements followed a similar trend, however significance was not reached (**Figure 1D**, bottom left). While Cr2 KO DZ area was still consistently lower than WT, a different profile was observed in comparison to LZ area with both strains peaking at day 6 before decreasing again at 14 days post immunization (**Figure 1D**, bottom right).

To confirm that Cr2 KO mice have impaired antibody production, serum samples were harvested from the same WT and Cr2 KO mice utilized in microscopy experiments. As expected, both IgM- and IgG-anti-SRBC antibodies were significantly reduced in Cr2 KO mice (**Figure 1E**).

Hence, analysis of 30-45 WP sections per group of mice, reveals that in our hands Cr2 KO mice have fewer and more rapidly declining GCs. The size of LZ and DZ increased with time in WT, but not Cr2 KO mice. These observations agree with previous results and suggest that the novel quantitative microscopy methodology used is adequate.

### Cr2 KO Mice Have Fewer FDCs in GCs Than WT Mice

To determine how FDCs, with or without expression of CR1/2, respond to SRBC immunization, we analyzed the presence of FDCs (FDC-M1+, blue) within B cell follicle, GC, LZ and DZ ROIs (as defined in **Figure 1B** **)**. Laser power and image acquisition settings were kept constant throughout experiments to allow for quantification of FDCs by the FDC-M1 mean fluorescence intensity (MFI) within WP regions only, thereby avoiding negative red pulp regions. WT, but not Cr2 KO, mice showed an increase in MFI from day 0 to day 6, suggesting a significant increase in FDC presence (**Figure 2A**). While no significant differences in FDC-M1 MFI were observed between WT and Cr2 KO mice, Cr2 KO mice consistently showed lower expression and returned to baseline at day 14 (**Figure 2A** **)**.

FDC areas were calculated by selecting FDC-M1 positive regions (thresholded), within various ROIs, i.e. excluding FDCs outside selected ROI for each calculation. FDC areas in the B cell follicle showed no significant differences between WT and Cr2 KO mice (**Figure 2B** ). The FDC area in Cr2 KO mice remained constant over time while the WT FDC area increased, peaking at day 3 post immunization before decreasing significantly to similar levels as in Cr2 KO mice at day 14 (**Figure 2B**).

FDC areas in the LZ of GCs was significantly larger at day 6 post immunization in WT than in Cr2 KO mice (**Figure 2C**, left). FDCs were traditionally thought to be present mainly in the LZ of GCs, but have also been identified in the DZ (3, 9). We successfully detected the presence of FDC-M1 expressing FDCs in the DZ in both WT and Cr2 KO mice (**Figure 2C**, right), thus confirming previous observations. While DZ FDCs were found in both WT and Cr2 KO mice, FDC area was significantly higher in WT mice, peaking at 6 days post immunization (**Figure 2C**, right). FDC areas in DZ and LZ were low in Cr2 KO mice. However, thresholding on unaltered images remained consistent between all groups, and FDC staining is evident in example images (**Figure 1B**). This indicates that these values are indeed above background and that FDCs are present in the DZ also of Cr2 KO mice, albeit at low levels.

Next, we wanted to investigate the proportion of the total FDC area that was located within GCs, LZ or DZ, or outside GCs (non-GC follicle area) (**Figure 2D** **)**. Follicles lacking GCs were excluded from analysis so as not to skew the percentage of FDCs located outside GCs, as illustrated by example images (**Figure 2E**). Overall, the majority of FDCs were located in the LZ in both WT and Cr2 KO mice. The proportion in the DZ increased at day 6 and 14 in comparison to day 3 post immunization, particularly in WT mice. However, the most notable difference was observed in the percentage of non-GC FDCs at day 3 and 6, with 5-9.4% of total FDCs in WT mice and 32.4-37.4% in Cr2 KO mice. Thus, not only are there fewer FDCs in Cr KO mice but they show different localization, even in GC positive splenic WP regions.

To ensure that computational analysis in **Figures 2B, C** did not bias results based on ROI selection, FDC colocalization with GC and follicle markers were also analyzed using whole fields of view, i.e. images without any ROI selection (**Supplementary Figure 4**). No difference between WT and Cr2 KO FDC colocalization with B220+ areas, i.e. follicles, was observed (**Supplementary Figure 4A**), thus supporting **Figure 2B**. WT FDCs showed significantly higher colocalization with Ki67+, i.e. GCs, at days 6 and 14 post immunization (**Supplementary Figure 4B**), thus supporting **Figure 2C**.

In summary, WT FDCs increased in number, as indicated by intensity (MFI) and FDC area, post SRBC immunization. FDCs were detected in LZ as well as DZ of both WT and Cr2 KO mice, although at much lower levels in Cr2 KO mice. Cr2 KO FDCs showed higher GC exclusion at earlier time points than WT FDCs.

### Expression of CR1/2 Is Needed for Directing FDCs to GCs

The observation of a substantial proportion of FDCs outside of the GC, “non-GC FDCs” (**Figures 2D, E**) was unexpected. It is important to consider that the GC does not have a distinct border as such. Hence, translating biological grey zones into computationally binary defined areas, e.g. GC ROIs, may lead to exclusion of FDCs which reside within the GC but close to the border (ROI edge). Therefore, the distance of non-GC classified FDCs from the GC border is essential in determining if the previously observed higher proportion of non-GC FDCs in Cr2 KO mice (**Figures 2D, E**) is truly reflective of biological differences. Distance maps of FDCs from ROI center or border were generated as described in Materials and Methods, and **Supplementary Figure 1**. Briefly, this was achieved by setting the center or edge of the desired ROI as the initiating point and increasing the intensity of each pixel by one as distance increases. Thereby, by overlaying FDC areas, a distance map is generated. The distance of non-GC FDCs from the GC border was significantly higher in Cr2 KO mice at days 6 and 14 post-immunization in comparison to WT mice (**Figure 3A**). This is in line with the findings in **Figure 2D** that non-GC classified FDCs in Cr2 KO mice are indeed distributed outside GCs at a higher proportion than non-GC FDCs from WT mice.

Differences were also observed between FDCs from the two strains within the GC ROI. FDCs from WT mice were consistently close to the GC center, while FDCs from Cr2 KO mice fluctuated in their distance from the center (**Figure 3B**). Closer inspection of LZ and DZ regions of GCs showed that both LZ and DZ FDCs in WT mice were further from LZ and DZ center (**Figures 3C, D**, left) and closer to LZ/DZ border (edge) (**Figures 3C, D**, right) than corresponding FDCs from Cr2 KO mice. This was particularly evident at day 14 post immunization (**Figures 3C, D**, right), at a time when GC numbers were significantly lower in Cr2 KO mice (**Figure 1C**). The LZ/DZ border typically divides the GC through, or close to, the GC center. Therefore, these observations correlate with observations that total GC FDCs in WT mice were more central than in Cr2 KO (**Figure 3B**). These differences in distance indicate that WT FDCs, but not Cr2 KO FDCs, move closer to LZ/DZ border when GCs are functioning at their peak and then separate when the response begins to dissipate.

Hence, FDCs from Cr2 KO mice are less confined to GCs, with non-GC FDCs localized further away from GC border than in WT mice. GC FDCs in WT mice remain close to GC center throughout the response while GC FDCs from Cr2 KO mice reside further away from the center, closer to LZ/DZ border (edge).

### Cr2 KO FDCs Show Different Organization to WT FDCs in Follicles, GCs, and LZ

In addition to determining how FDCs were located in relation to the center and border of whole GCs, LZ and DZ (**Figure 3**), we wanted to establish how they are organized within these structures. To determine whether FDCs are randomly or non-randomly distributed, proximity of FDC areas to one another were analyzed. This was achieved by measuring the rate at which follicle, GC or LZ ROIs were filled by the repeated computational dilations of FDC areas (**Figure 4A**) (37) and comparing the fill rate to that of a randomized control. This method measures the proximity of FDC areas to one another within the follicle, GC or LZ. The FDC areas are repeatedly dilated, sequentially adding 1 pixel to each FDC area and the cumulative area of all FDC areas recorded. Dilation continues until the entire ROI is filled (i.e. 100%). The randomized controls were generated using the RandomJ ImageJ plugin in which individual FDC areas were randomly distributed within the selected ROI eight times and averaged for each sample image. When FDC areas are clustered, the dilations from nearby FDC areas quickly overlap and therefore the rate at which the ROI is filled is slow. When FDC areas are dispersed, their separation delays the merger of dilated areas and thereby speeds up the ROI filling. This is then plotted as the percentage of total ROI filled after each dilation (solid line) and compared to randomized control (dotted line). This allows FDC clustering (reduced fill rate, lower than random) or actively dispersing (a faster fill rate, above random) to be quantified (**Figure 4A**).

FDCs within the entire B cell follicle was studied as a proof-of-concept exercise. Given that FDCs reside mainly within GCs, FDCs were expected to cluster within follicle ROIs as GCs are formed. Indeed, follicle FDCs in WT mice consistently showed clustering, even in naive mice (day 0) (**Figure 4B**). Increased clustering, indicated by larger differences from randomized controls, were observed at day 6 and day 14 post SRBC immunization but was only significant at day 6 (**Figure 4B**). In contrast, follicle FDCs from Cr2 KO mice showed randomized organization at all times (**Figure 4B**). This reflects the expected clustering into GCs of follicular WT FDCs, both in the resting, non-immunized, state and, more pronounced, at the peak of the GC response day 6.

Confining the analysis to GCs, WT FDCs showed significant active dispersion at all time points, while in Cr2 KO mice GC FDCs were dispersed at day 3 but clustered at days 6 and 14 (**Figure 4C**). It may seem paradoxical that WT FDCs are clustered in the follicle (**Figure 4B**) but dispersed in the GC (**Figure 4C** **)**. However, the clustering in the follicle merely indicates that FDCs, as expected, are located in the GCs within the follicle - GCs are per definition clusters. On the other hand, the organization of FDCs in a GC is indicative of how they react within this area. Organization of LZ FDCs (**Figure 4D**) gave similar results as the organization of total GC FDCs (**Figure 4C**). Overall LZ and GC FDCs from both strains organized similarly within the respective strain. An exception is seen at day 6 post immunization when LZ FDCs show greater separation (i.e. active dispersion) than GC FDCs in both WT and Cr2 KO. This indirectly suggests that DZ FDCs are less separated (i.e. more clustered) than LZ FDCs.

In summary, GC FDCs in WT mice were actively dispersed, while GC FDCs in Cr2 KO mice were either randomly organized or clustered. Overall, few differences were observed between total GC FDC and LZ FDC organization. Interestingly, the dispersion of WT FDCs in the LZ increased with time after immunization with a maximum at day 6.

### Expression of CR1/2, FcγRIIB, and FcμR Increases in Response to SRBC in WT Mice

To observe changes in CR and FcR expression in FDCs over the 14 day time course after SRBC-immunization, spleen sections were stained for FDCs (FDC-M1, blue, and CR1, grey) and various receptors (green): CR1/2 (**Figure 5A**, **Supplementary Figure 5**), FcγRIIB (**Figure 5B**, **Supplementary Figure 6**), or FcµR (**Figure 5C**, **Supplementary Figure 7**). Colocalization of FDCs with receptors (M1) and, most importantly, colocalization of receptors with FDCs (M2) by Mander’s colocalization coefficients were calculated (**Figures 6A–F** **)**.

As CR1 is commonly used as a marker for FDCs, high consistent colocalization between FDC-M1 and CR1 was as expected (**Figure 6A**). Similar results were obtained with CR1/2, which showed stable expression on WT FDCs with the exception of day 14 post immunization where a significant increase was detected (**Figure 6B**). As expected CR1 and CR1/2 was not detected in Cr2 KO mice. FcγRIIB was elevated at day 14 in WT but not Cr2 KO mice when FDC-M1 was used to stain FDCs (**Figure 6C**). Similarly, FcγRIIB day 14 upregulation was observed using CR1 as the marker for FDCs (**Figure 6E**). Interestingly, expression of FcµR was also significantly increased at day 14 in WT FDCs compared to either day 0 (using FDC-M1 as the FDC marker; **Figure 6D**) or to day 1 (using CR1 as the FDC marker; **Figure 6F**). The observation that FDCs express FcµR is novel and therefore FDCs expression of FcµR was confirmed by flow cytometry (**Supplementary Figure 8**).

In order to compare receptor expression on all immune cells within the WP ROI and not just FDCs, MFIs of WT and Cr2 KO mice were analyzed at day 14 post immunization (**Figure 6G**). WT mice showed significantly higher expression of CR1, CR1/2, FcγRIIB and FcμR. However, as B cells also express these receptors, this increase cannot be correlated solely with FDCs.

Thus, expression of CR1/2, FcγRIIB and FcµR increased in WT mice at day 14 post immunization. In contrast, neither expression of FcγRIIB nor FcµR increased in Cr2 KO mice. As expected, these mice lacked expression of CR1 and CR1/2.

## Discussion

FDCs are notoriously difficult cells to isolate and culture, and many details on how they behave during an immune response remain elusive. Here, we have developed a simple, yet powerful, mathematical method to analyze numbers, distribution and organization of FDCs. This technique enables detailed comparison of GC dynamics using unbiased computational analysis of spleen sections. We employed this novel method to study the behavior of FDCs in WT mice and to investigate whether expression of CR1/2 on FDCs affected their behavior over a 14 day SRBC immunization time course.

This experimental model was selected because SRBC induce a strong antibody and splenic GC response after administration without adjuvants and because these responses are dependent on CR1/2 (reviewed in (15, 16). These receptors are expressed on both B cells and FDCs (18) and studies in bone marrow chimeras have implicated an important role of CR1/2 on FDCs (25, 26, 28–30), including in responses to SRBC (25, 28). As expected, IgM and IgG responses as well as GC responses were impaired in Cr2 KO mice (**Figure 1** **)**. This was in line with previous observations and confirmed that analyzing GCs with our approach gives comparable results. Having established this, we performed detailed studies of FDCs and their localization within follicles and GCs, including DZ and LZ distribution.

An interesting finding was that a substantial proportion of FDCs in Cr2 KO mice resided in B cell follicles outside of GCs (**Figures 2** and **3**). Defining the GC/follicle border for binary computational analysis may result in FDCs at the biological GC border being excluded from the computationally defined GC ROI. Although ROI definitions were consistent between strains and groups, this putative margin of error must be considered when evaluating the data. As non-GC FDCs in WT mice were located close to the GC/follicle border, this margin of error may account for a large proportion of the relatively few non-GC FDCs observed in this strain. Assuming that this is the case, the percentage of non-GC FDCs would be lower than observed. Non-GC FDCs in Cr2 KO mice were further from the GC/follicle border and their high proportion is therefore unlikely to be explained by any margin of error. Thus, it is possible that the difference in proportion of non-GC FDCs between the two strains may be even bigger than indicated. In summary, these observations show that FDCs are present in follicles even when GCs are scarce, and suggest that FDCs are recruited from these areas upon the formation of GCs.

From the present studies, it is not possible to directly determine whether CR1/2 expression on FDCs and/or B cells are responsible for the impaired recruitment of CR1/2-deficient FDCs to GCs. However, using the same Cr2 KO and WT strains to create bone marrow chimeras, immunizing them with the same dose of antigen used herein (5 x 10^7^ SRBC), it was shown that IgG anti-SRBC responses were extremely dependent on expression of CR1/2 on FDCs, but not on B cells (28). Therefore, it is likely that the effects of CR1/2 on FDCs and GC formation shown here are largely dependent on FDC expression of the receptors. However, a role for CR1/2-deficient B cells cannot be excluded. Likewise, it cannot be excluded that the relative involvement of B cells and FDCs *via* CR1/2 varies depending on antigen and immunization protocols, e.g. whether adjuvants were used or not. Recruitment of FDCs into GCs relies on lymphotoxin (LT), and maturation relies on both LT and tumor necrosis factor (TNF), which are produced by B cells (38, 39). LT and TNF KO mice both have a significant reduction of FDCs (40, 41). Similarly, Cr2 expression was strongly inhibited in Rag1 KO mice, indicating a key role of B cells in FDC maturation (4). While B cell numbers are largely similar in WT and Cr2 KO mice (24, 32), lack of CR1/2 expression may cause reduced B cell activation owing to decreased signaling (19, 20), and thus hamper the cytokine gradients essential for establishing GC regions within secondary lymphoid organs. This change in survival and homing signals is a possible explanation for the reduced number of FDCs within GCs and the high proportion of non-GC FDCs observed in Cr2 KO mice. This may also explain why non-GC FDCs localize further from the GC/follicle border in Cr2 KO than in WT mice.

Cr2 KO mice have less FDCs post SRBC immunization than WT mice. This is demonstrated by reduced MFI in WP and the pronounced differences between FDC-M1 positive areas in the LZ and DZ of GCs (**Figure 2**). Although the majority of FDCs were found in the LZ, DZ FDCs were also detected in both strains. DZ FDCs were recently identified to be a subset of FDCs that produce high levels of CXCL12 (9). Their expression of CR1/2 was lower than in LZ FDCs, as was expression of many FcRs (9). This suggests that DZ FDCs do not present ICs as efficiently as LZ FDCs but assist in setting up the topological remodeling of LZ and DZ regions. Overall, FDCs in WT mice were more centrally located within the total GC ROI than FDCs in Cr2 KO mice, indicating that CR1/2 is required for the ability of FDCs to localize in an efficient manner.

Previous studies into GC cytokine gradients and their role in GC topography suggest that the CXCL12-dependent organization of LZ and DZ FDCs determines the efficacy of humoral immunity by steering the interaction of B and T cells in the GC (9). Hence, FDC organization may affect overall GC topography. To address this, organization of FDCs in the two strains was also observed relative to randomized controls in follicles and GCs (**Figure 4**). WT follicle FDCs showed clustered distribution, most likely reflecting that FDCs form GCs during the immune response. A clustered distribution was observed even in naive WT mice (day 0), possibly reflecting the spontaneous GCs sometimes observed in the spleen. In comparison, FDCs in follicles of Cr2 KO mice were randomly distributed and never showed significant clustering, indicating that the FDC network throughout the B cell follicle in part relies on complement and CRs for optimal organization. Confining the analysis to GCs, FDCs in Cr2 KO mice exhibited either random or clustered organization, while FDCs in WT mice were actively dispersed. The consistent dispersion of WT FDCs in GCs may have biological significance in allowing higher surface area for B cell interaction in comparison to the more clustered Cr2 KO FDCs.

FDCs express a variety of receptors able to capture ICs: CR1, CR2, FcϵRII, FcγRIIB and Fcα/µR (5–12). We found that expression of CR1/2, FcγRIIB and FcμR increased significantly in WT mice at day 14 after SRBC-immunization while no increase in FcγRIIB or FcμR expression was seen in Cr2 KO mice (**Figures 5** and **6**). This indicates that WT, but not Cr2 KO FDCs, become activated during the immune response. FcγRIIB has been well documented as an important receptor in propagation of FDC antigen presentation (1, 5–7, 9) and it is no surprise that WT FDCs increased their expression of FcγRIIB. FcμR has previously been observed to be expressed by B cells but not myeloid cells (42, 43). Therefore, it is very interesting that FcμR was detected on activated WT FDCs, both by microscopy and by flow cytometry. Our results add FcµR to the already known receptors on FDCs which are able to capture ICs containing IgM and complement: CR1, CR2, and Fcα/µR. This high redundancy suggests that capture of ICs formed early during an immune response, before IgG is produced and FcγRIIB can be engaged, is an important function of FDCs.

The importance of FDCs, and their expression of CR1/2, in humoral immune responses has classically been attributed to their ability to capture and present complement-containing ICs. In addition, it is known that Cr2 KO mice often have impaired development of GCs, a finding we confirm using computational analysis. Utilizing our novel computational ImageJ macro analysis, we have here described several additional factors that may decrease the ability of FDCs lacking CR1/2 to support humoral immune responses. During the first two weeks of an immune response, FDCs in the immune deficient Cr2 KO mice (i) are fewer than in WT mice, (ii) do not increase expression of FcγRIIB or FcµR, (iii) are to a large extent localized outside of GCs, and (iv) exhibit a clustered rather than a dispersed organization within the GCs.

## Data Availability Statement

The original contributions presented in the study are included in the article/**Supplementary Material**. Further inquiries can be directed to the corresponding authors.

## Ethics Statement

The animal study was reviewed and approved by Uppsala Animal Research Ethics Committee.

## Author Contributions

JCA, JA, and BH designed the study. JCA and AW performed the experiments. JCA, BH, and JA wrote the manuscript. All authors contributed to the article and approved the submitted version.

## Funding

This work was supported by Uppsala University, The Swedish Research Council (#2015-02605 and 2018-02409), Olle Engkvist Byggmästare Foundation, King Gustaf V:s 80 Years Foundation (FAI-2017-0374), Gurli and Edward Brunnberg Foundation, and Agnes and Mac Rudberg’s Foundation. 

## Conflict of Interest

The authors declare that the research was conducted in the absence of any commercial or financial relationships that could be construed as a potential conflict of interest.

## Abbreviations

CRs, complement receptors; EDM: Euclidean Distance Map; F, B cell follicle; FDCs, follicular dendritic cells; FcRs, Fc Receptors; GCs, germinal centers; ICs, immune complexes; ROIs, regions of interest; SRBCs, sheep red blood cells; WP, white pulp.
