# Supplementary material for: A Novel Image Analysis Approach Reveals a Role for Complement Receptors 1 and 2 in Follicular Dendritic Cell Organization in Germinal Centers
Source: Front Immunol. 2021 Apr 12;12:655753. doi: 10.3389/fimmu.2021.655753 (PMC8072117; doi:10.3389/fimmu.2021.655753)
Supplement: Supplementary file 1 [file DataSheet_1.pdf]

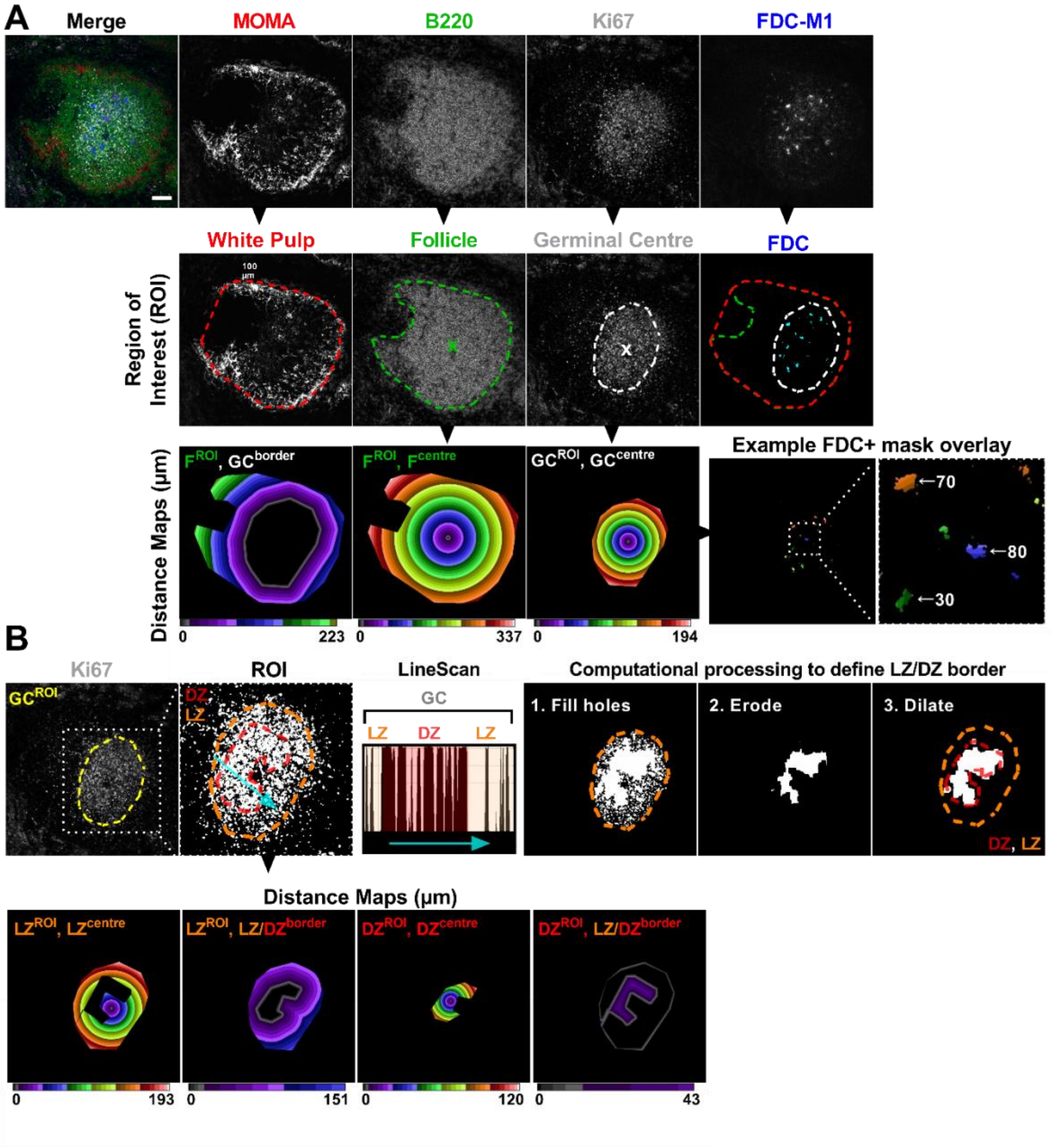

Supplementary Figure 1. Outline of image analysis workflow for (A) White pulp (WP) and **(B)** GC regions. Original unaltered images were split into individual channels to allow identification of white pulp regions (surrounded by MOMA+ metallophilic macrophages (red)), B cell follicle (B220+ B cell regions (green) and germinal centers (concentrated Ki67 regions (grey)). **(A)** White pulp regions of interest (ROIs) and **(B)** GC regions, which were subdivided into light (LZ) and dark (DZ) GC regions based on Ki67 density in thresholded images. The computational processing by which the LZ/DZ ROIs were determined are illustrated as a threestep process where holes in the thresholded Ki67+ image, then these ROIs are eroded to eliminate small (LZ) ROIs and then dilated to conserve the remaining (DZ) ROI area. The center point of each ROI was utilized to generate distance maps (Euclidean Distance Map (EDM), (Follicle (F^{center}), GC (GC^{center}), LZ (LZ^{center}), DZ (DZ^{center}). Distance maps were confined

to different ROIs (Follicle (F^{ROI}), GC (GC^{ROI}), LZ (LZ^{ROI}), DZ (DZ^{ROI}). In addition, EDM distance maps were generated to measure distance from the GC border (GC^{border}), and LZ/DZ border (LZ/DZ^{border}). FDCs detected by FDC-M1 (blue) were thresholded and positive regions overlayed by respective distance maps. FDC area in each region and distances were plotted. Average area and distances were subsequently analyzed in Figs 1-4.
