# Supplementary material for: A Novel Image Analysis Approach Reveals a Role for Complement Receptors 1 and 2 in Follicular Dendritic Cell Organization in Germinal Centers
Source: Front Immunol. 2021 Apr 12;12:655753. doi: 10.3389/fimmu.2021.655753 (PMC8072117; doi:10.3389/fimmu.2021.655753)
Supplement: Supplementary file 2 [file DataSheet_2.pdf]

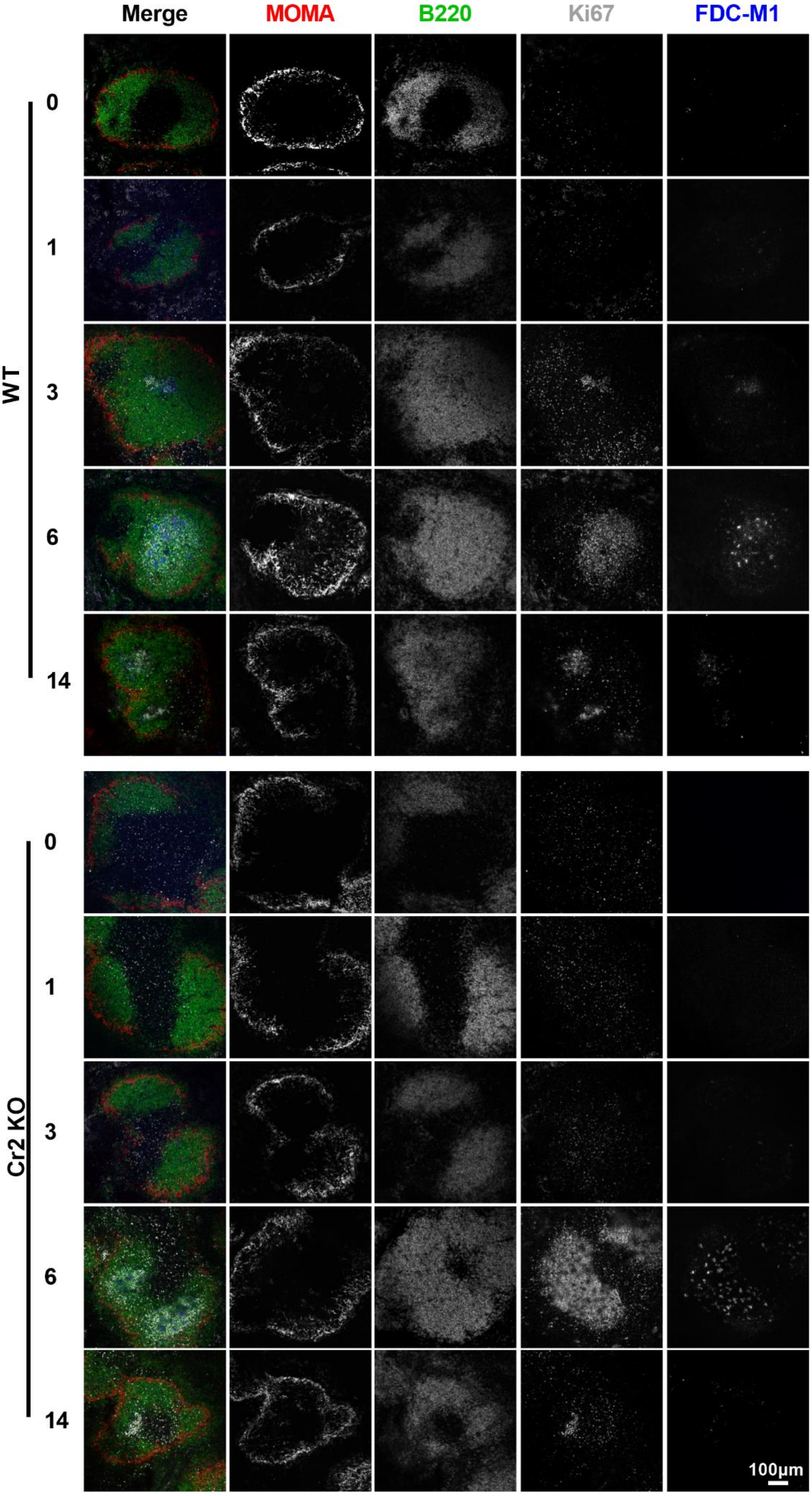

Merge
MOMA
B220
Ki67
FDC-M1
WT
0
1
3
6
14
Cr2 KO
0
1
3
6
14
100µm

Supplementary Figure 2. Expanded representative images of spleen white pulp regions. WT or Cr2 KO mouse spleens and sera were harvested from unimmunized mice (day 0) and from mice immunized with 5 x 10^7 SRBC 1, 3, 6, or 14 days before and sections prepared as described in Materials and Methods. Expanded representative images of splenic white pulp regions (surrounded by MOMA+ metallophilic macrophages, red), B cell follicles (B220, green), GCs (Ki67, grey) and FDCs (FDC-M1, blue). Data shown is representative of 6-9 mice per group pooled from three independent experiments.
