# Supplementary material for: A Novel Image Analysis Approach Reveals a Role for Complement Receptors 1 and 2 in Follicular Dendritic Cell Organization in Germinal Centers
Source: Front Immunol. 2021 Apr 12;12:655753. doi: 10.3389/fimmu.2021.655753 (PMC8072117; doi:10.3389/fimmu.2021.655753)
Supplement: Supplementary file 3 [file DataSheet_3.pdf]

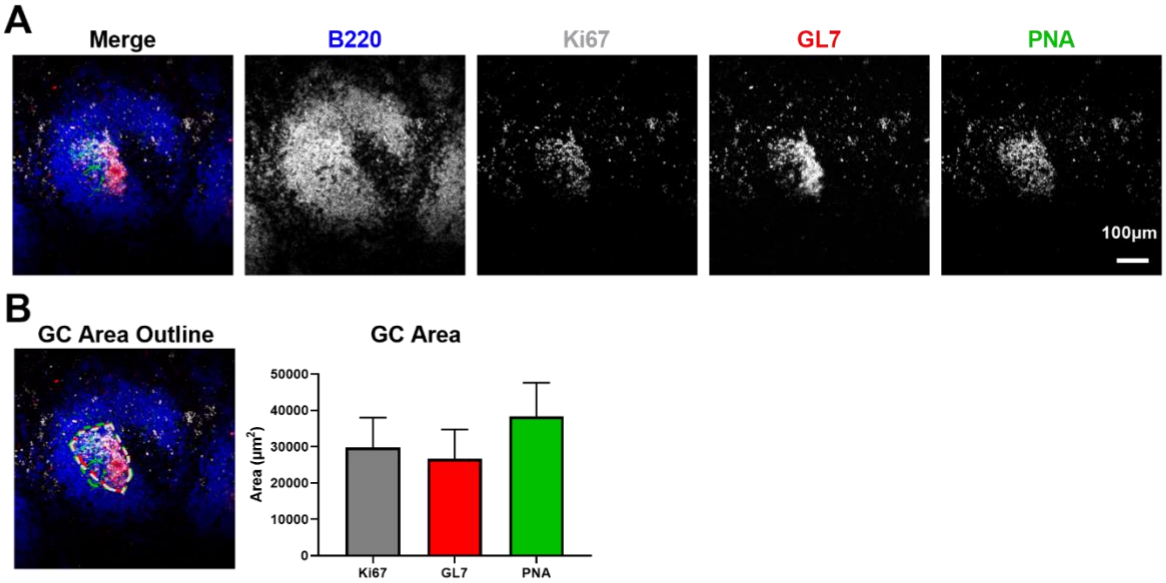

Supplementary Figure 3. Germinal center staining comparison. (A) Expanded representative images of spleen B cell follicle (B220, blue), containing a GC stained with Ki67 (grey) GL7 (red) and PNA (green). **(B)** Example GC ROI area outline of Ki67+ area (grey line), GL7+ area (red line) and PNA+ area (green line). GC area (μ2 ± SEM) based on each GC marker. The data is representative of 3 mice from two independent experiments. Statistical differences between GC markers in B were determined by two-way ANOVA. *** p values< 0.001, ** < 0.01, and * < 0.05.
