# Supplementary material for: A Novel Image Analysis Approach Reveals a Role for Complement Receptors 1 and 2 in Follicular Dendritic Cell Organization in Germinal Centers
Source: Front Immunol. 2021 Apr 12;12:655753. doi: 10.3389/fimmu.2021.655753 (PMC8072117; doi:10.3389/fimmu.2021.655753)
Supplement: Supplementary file 4 [file DataSheet_4.pdf]

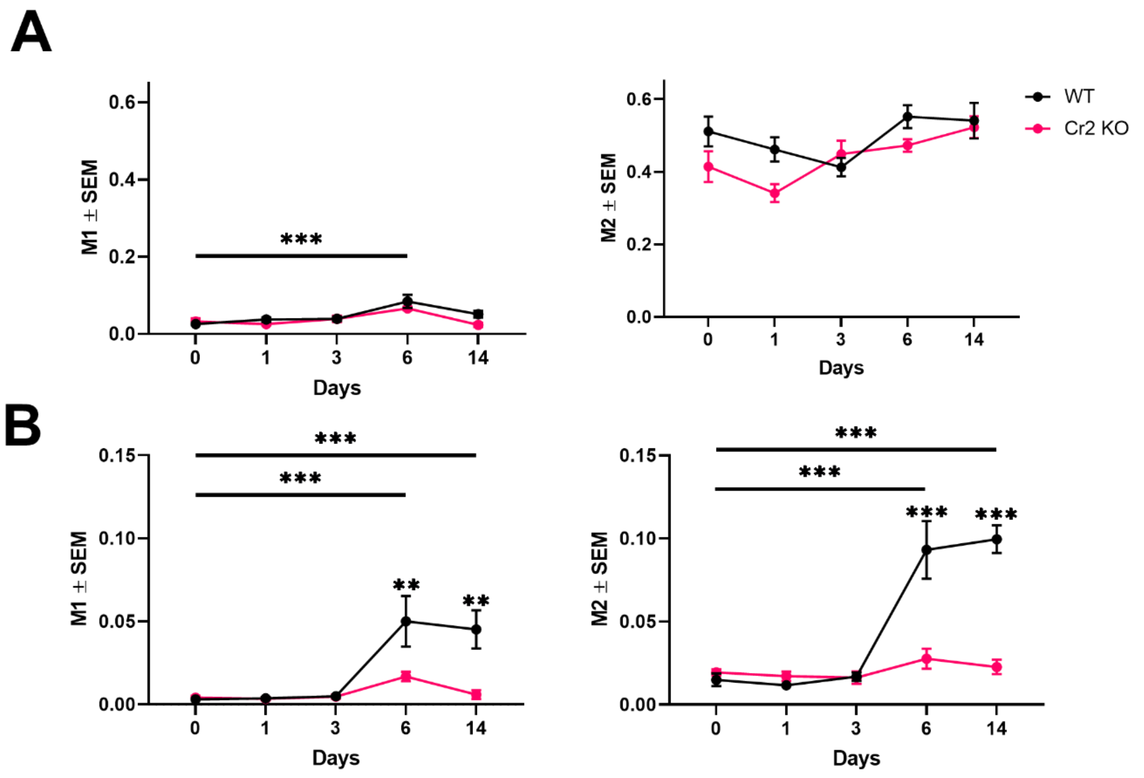

Supplementary Figure 4. FDCs are located in germinal centers. WT or Cr2 KO mouse spleens and sera were harvested from unimmunized mice (day 0) and from mice immunized with 5 x 10^7 SRBC 1, 3, 6, or 14 days before and sections prepared as described in Materials and Methods. Mander's colocalization co-efficient of WT (black) and Cr2 KO (red) FDCs (FDC-M1) with **(A)** B cell follicle (B220) **(B)** GC (Ki67). M1 represents the colocalization of follicle or GCs with FDCs (frequency of FDCs in follicle or GC) and M2 reciprocally represents the colocalization of FDCs with follicle or GCs (frequency of follicle or GC where there are FDCs. Statistical differences between the groups were determined by two-way ANOVA. Statistical differences between WT and Cr2 KO mice at each time point is shown above the upper curve and statistical differences between naive and immunized respective mice over the 14 day time course are indicated above horizontal lines (WT= black; KO= red). *** p values< 0.001, ** < 0.01, and * < 0.05. The data shown are 6-9 mice per group pooled from three independent experiments.
