# Supplementary material for: A Novel Image Analysis Approach Reveals a Role for Complement Receptors 1 and 2 in Follicular Dendritic Cell Organization in Germinal Centers
Source: Front Immunol. 2021 Apr 12;12:655753. doi: 10.3389/fimmu.2021.655753 (PMC8072117; doi:10.3389/fimmu.2021.655753)
Supplement: Supplementary file 6 [file DataSheet_6.pdf]

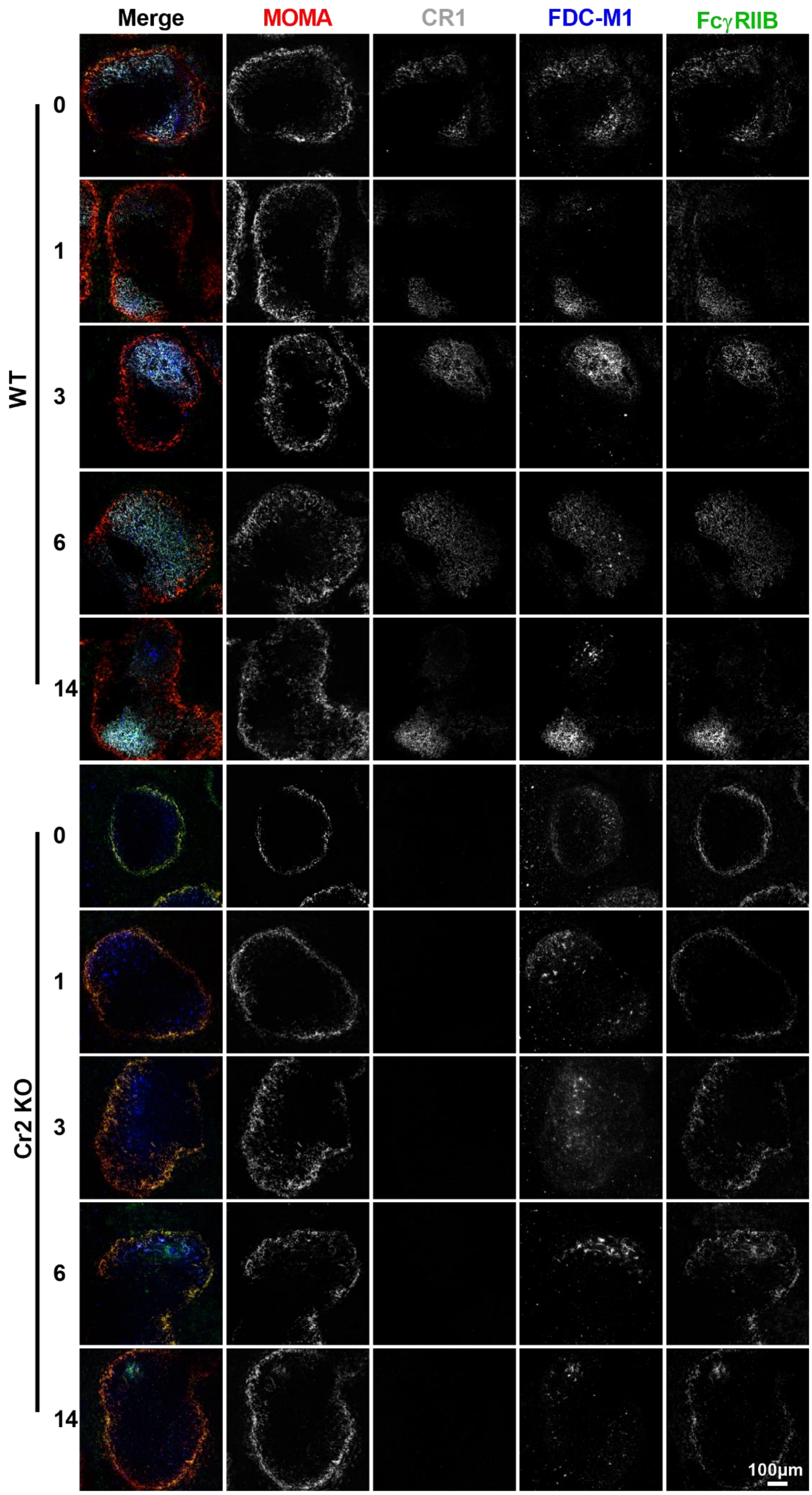

Merge
MOMA
CR1
FDC-M1
FcγRIIB
WT
0
1
3
6
14
Cr2 KO
0
1
3
6
14
100µm

Supplementary Figure 6. Expanded representative images of FcγRIIB expression in white pulp. WT or Cr2 KO mouse spleens and sera were harvested from unimmunized mice (day 0) and from mice immunized with 5 x 10^7 SRBC 1, 3, 6, or 14 days before and sections prepared as described in Materials and Methods. Representative merged and single channel images of SRBC immunized (naive (0), day 1-14) WT and Cr2 KO spleen where white pulp regions (surrounded by MOMA+ metallophilic macrophages, red), FDCs (FDC-M1, blue), CR1 (8C12, grey) and FcγRIIB (clone 93, green) are shown. Data shown is representative of 6-9 mice per group pooled from three independent experiment.
