# Supplementary material for: A Novel Image Analysis Approach Reveals a Role for Complement Receptors 1 and 2 in Follicular Dendritic Cell Organization in Germinal Centers
Source: Front Immunol. 2021 Apr 12;12:655753. doi: 10.3389/fimmu.2021.655753 (PMC8072117; doi:10.3389/fimmu.2021.655753)
Supplement: Supplementary file 7 [file DataSheet_7.pdf]

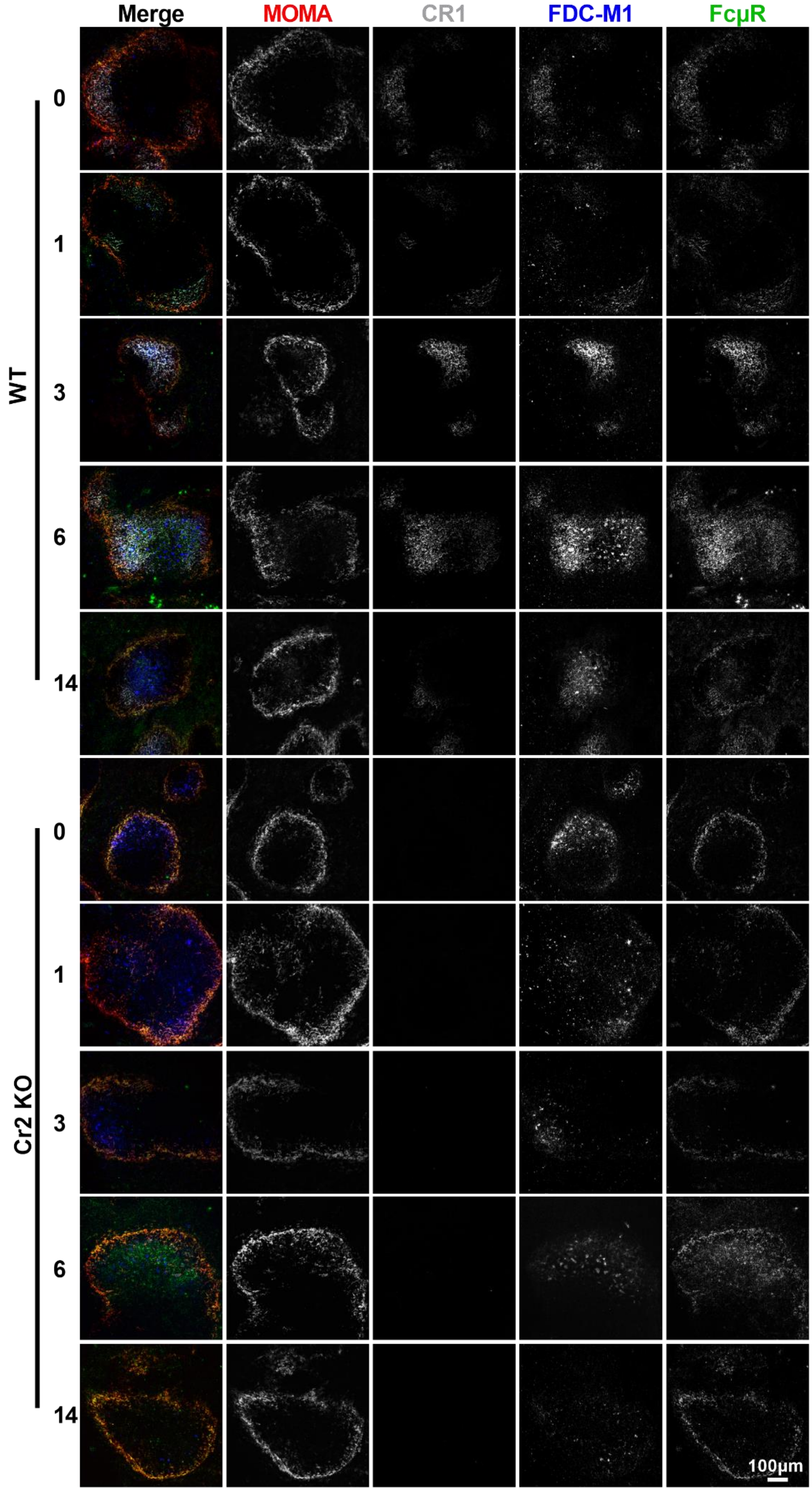

Merge
MOMA
CR1
FDC-M1
FcμR
WT
0
1
3
6
14
Cr2 KO
0
1
3
6
14
100μm

**Supplementary Figure 7. Expanded representative images of FcμR expression in white pulp.** WT or Cr2 KO mouse spleens and sera were harvested from unimmunized mice (day 0) and from mice immunized with 5 x $10^7$ SRBC 1, 3, 6, or 14 days before and sections prepared as described in Materials and Methods. Representative merged and single channel images of SRBC immunized (naive (0), day 1-14) WT and Cr2 KO spleen where white pulp regions (surrounded by MOMA+ metallophilic macrophages, red), FDCs (FDC-M1, blue), CR1 (8C12, grey) and FcμR (MM3, green) are shown. Data shown is representative of 6-9 mice per group pooled from three independent experiment.
