# Supplementary material for: A Novel Image Analysis Approach Reveals a Role for Complement Receptors 1 and 2 in Follicular Dendritic Cell Organization in Germinal Centers
Source: Front Immunol. 2021 Apr 12;12:655753. doi: 10.3389/fimmu.2021.655753 (PMC8072117; doi:10.3389/fimmu.2021.655753)
Supplement: Supplementary file 8 [file DataSheet_8.pdf]

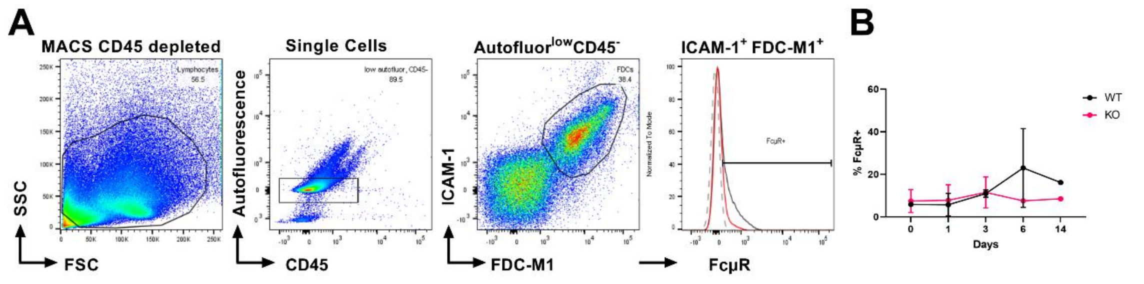

Supplementary Figure 8. Flow cytometry detection of FcμR expression on FDCs. WT or Cr2 KO mouse spleens and sera were harvested from unimmunized mice (day 0) and from mice immunized with 5 x 10^7 SRBC 1, 3, 6, or 14 days before and spleen single cell suspensions prepared as described in Materials and Methods. **(A)** Gating strategy and representative histogram of FDCs isolated from CD45 MACs depleted spleen single cell suspensions 6 days post SRBC immunization. Single cells were gated to exclude high autofluorescence (FITC channel) and CD45 (BV480) positive cells. FDCs were subsequently identified by high FDC-M1 (APC Cy7) and ICAM-1 expression (Alexa Fluor 545). The percentage of FDCs expressing FcμR (clone MM3, Alexa Fluor 594) in WT (black) and Cr2 KO (red) were then gated in comparison to fluorescence minus one (FMO) control (grey, broken line). **(B)** Percentage of FcμR positive FDCs isolated from SRBC immunized (naive (0), day 1-14 post immunization) WT and Cr2 KO mice. The data shown are 6 mice per group pooled from two independent experiments, where 3 mice were pooled per experiment.
